# Evaluation of the Safety of Dexmedetomidine Dosing Utilizing Adjusted Body Weight in Obese Critically Ill Patients: A Retrospective Study

**DOI:** 10.7759/cureus.81155

**Published:** 2025-03-25

**Authors:** Molly E Robertson, Aimee E Willett, Joshua J Mathias, Britney N Sleeckx, John O Elliott

**Affiliations:** 1 Pharmacy, OhioHealth Mansfield Hospital, Mansfield, USA; 2 Internal Medicine, OhioHealth Riverside Methodist Hospital, Columbus, USA; 3 Medical Education, OhioHealth Research Institute, Columbus, USA

**Keywords:** bradycardia, critical care, critically ill, dexmedetomidine, hypotension, obesity, pharmacology

## Abstract

The purpose of this study was to evaluate the safety of dexmedetomidine dosing, utilizing adjusted body weight (AdjBW) in obese, critically ill patients. This was a retrospective cohort study of patients who received dexmedetomidine from March 2020 to April 2021. Participants received dexmedetomidine as the sole agent for sedation for ≥8 hours, were ≥18 years old, had a level of care listed as “critical care,” and had an actual body weight (ABW) of at least 120% of their ideal body weight (IBW). A total of 225 participants were included. Results demonstrated that the incidence of hypotension and bradycardia was lower in the AdjBW group compared to the ABW group, but this did not reach statistical significance. Dosing dexmedetomidine based on AdjBW, instead of ABW, resulted in a statistically significant difference in the lowest recorded heart rate, with 61.2 ± 11.8 bpm in the ABW group and 65.2 ± 14.7 bpm in the AdjBW group (p = 0.027). Dosing dexmedetomidine based on AdjBW did not show statistically significant differences in the lowest recorded mean arterial pressure (MAP). Using AdjBW to dose dexmedetomidine appears to be safe in this patient population.

## Introduction

Obesity affects approximately 20% of all critically ill patients admitted to the hospital, and it can alter the pharmacokinetic properties of many drugs, including those used for sedation [1]. Critically ill patients tend to have altered absorption, distribution, metabolism, and elimination, which can lead to either toxic or suboptimal drug concentrations [2]. The fat mass may affect drug concentrations through impaired clearance and volume of distribution changes [3]. Optimal dosing strategies are limited in this patient population due to the lack of evidence from supporting literature. Many of the commonly used medications for sedation lack labeling for dosing in obese patients [4]. Dexmedetomidine is a highly selective α2-adrenoreceptor agonist that produces dose-dependent sedation and anxiolysis, with a lower incidence of respiratory depression compared to other sedative agents [5]. Two of the most common side effects of dexmedetomidine include hypotension and bradycardia [6]. Previous studies compared dexmedetomidine dosing based on actual body weight (ABW) in obese patients to normal-weight patients in the perioperative setting. Obese patients had increased sedation, lower oxygen saturation, and higher drug concentrations compared to those of normal weight [3,7]. Atyia et al. concluded that dosing dexmedetomidine using adjusted body weight (AdjBW) in obese, critically ill patients, compared to ABW, resulted in no difference in the percent of Richmond Agitation Sedation Scale (RASS) measurements within goal [1]. This showed that lower doses of dexmedetomidine were still effective at maintaining light to moderate sedation in this patient population.

Our institution recently implemented a protocol change for dosing dexmedetomidine using AdjBW instead of ABW, unless ABW is less than ideal body weight (IBW). This was implemented to limit the incidence of bradycardia and hypotension from increased doses of dexmedetomidine, when lower doses have been shown to be as effective. The dexmedetomidine dosing protocol at our institution begins at 0.4 mcg/kg/hr for mechanically ventilated patients, or 0.2 mcg/kg/hr for non-mechanically ventilated patients, and is titrated by RASS goal by no more than 0.2 mcg/kg/min every 15 minutes, up to 1.5 mcg/kg/hr. If hypotension or bradycardia develops while on infusion, nursing is to notify the physician for instructions to titrate the infusion.

The purpose of this study was to evaluate the impact of AdjBW dexmedetomidine dosing on the occurrence of hypotension and bradycardia in obese, critically ill patients, following the protocol change at our institution. We hypothesized that side effects of hypotension and bradycardia would be more prevalent in the ABW group, compared with the AdjBW group.

## Materials and methods

Study design, setting, and participants

This was a retrospective cohort study of obese, critically ill patients who received dexmedetomidine across a single hospital system in central Ohio. Potential study subjects were identified via a query of an existing institutional database and included critically ill patients across medical, surgical, neurocritical, and cardiovascular intensive care units, requiring sedation with dexmedetomidine, with an ABW greater than 120% of their IBW. The inclusion criteria were age ≥18 years old, level of care listed as “critical care” in the electronic medical record (EMR), weight ≥120% of IBW, and receiving dexmedetomidine for ≥8 hours as the sole agent for sedation. Patients were excluded if they had a RASS goal less than -2, were pregnant, had a positive COVID-19 test at any point during their hospital admission, or had a pacemaker. Patients with duplicate admissions were not included in this study.

Study timeline and intervention

Patients were divided into two groups based on the dosing weight of dexmedetomidine and the timeframe in which their encounter occurred. Patients in group 1 were admitted from March 14, 2020, to September 20, 2020, and they received dexmedetomidine dosing based on their ABW. Patients in group 2 were admitted from October 19, 2020, to April 19, 2021, and they received dexmedetomidine dosing based on their AdjBW. A washout period, from September 21, 2020, to October 18, 2020, was included. The live environment for the protocol change was October 5, 2020, so no data were collected during this time period to ensure patients were not included in more than one group.

The preliminary patient list generated from this query was reviewed and validated by the investigators or delegated study staff, to ensure an accurate data pull and to confirm that all patients met inclusion/exclusion criteria.

Outcomes

The primary outcome was to compare the incidence of hypotension and bradycardia in obese, critically ill patients receiving dexmedetomidine based on their ABW versus AdjBW. Hypotension was defined as a mean arterial pressure (MAP) less than 65 mmHg, and bradycardia was defined as a heart rate less than 55 beats per minute. The secondary outcomes were to compare the number of patients in the ABW group versus the AdjBW group who required additional sedatives, the incidence of self-extubation among intubated patients, hospital length of stay, lowest recorded MAP, and lowest recorded heart rate between the two groups. Additional sedative continuous infusions included propofol, opioids, ketamine, and benzodiazepines.

Power analysis and sample size calculation

Literature estimates overall rates of hypotension of 41% versus 57% in the ABW group and the AdjBW group, respectively [1]. This study estimated a sample size of 306 patients (153 in each group), with a power set at 80% and an alpha rejection value set at 5%, to detect a statistically significant difference between the two groups.

Statistical analysis

Univariate comparisons of demographic and clinical factors for the two treatment groups (obese patients receiving dexmedetomidine based on either AdjBW or ABW) were made using independent sample t-tests (both equal variances assumed and equal variance not assumed) for continuous data, and Chi-square tests for categorical data. If the data were skewed, the Wilcoxon rank sum test was also reported.

## Results

Patient demographics

A total of 302 patients were included for analysis between March 14, 2020, and April 19, 2021. There were 77 patients excluded for having improper or missing documentation upon manual chart review, which left a total of 225 patients for the analysis (98 patients in the ABW group and 127 patients in the AdjBW group). Patient demographics and clinical characteristics were similar between the two groups (Tables 1-2).

**Table 1:** Patient Demographics *p-value based on the Wilcoxon rank sum test Chi-square tests are used for nominal data, and independent samples t-tests are used for continuous data BMI: body mass index

Characteristic	Actual Body Weight (n = 98)	Adjusted Body Weight (n = 127)	Test Statistic	p-value
Age, Mean ± SD	62.2 ± 14.3	63.1 ± 15.0	0.462	0.645
Sex, n (%)			0.775	0.420
Female	49 (50.0)	56 (44.1)	-	
Male	49 (50.0)	71 (55.9)		
Race, n (%)			1.497	0.683
African American Participants	7 (7.1)	9 (7.1)	-	
American Indian or Alaska Native Participants	0 (0)	0 (0)		
Asian Participants	1 (1.0)	0 (0)		
Caucasian Participants	85 (86.7)	113 (89.0)		
Declined to Specify	5 (5.1)	5 (3.9)		
Ethnicity, n (%)			3.772	0.152
Declined	0 (0)	4 (3.2)	-	
Hispanic or Latino	2 (2.0)	1 (0.8)		
Not Hispanic or Latino	96 (98.0)	121 (96.0)		
BMI (kg/m^2^), Mean ± SD	37.5 ± 10.6	35.7 ± 9.2	1.376	0.170
Actual Body Weight (kg), Mean ± SD	107.7 ± 30.4	102.4 ± 30.0	1.326	0.186
Adjusted Body Weight (kg), Mean ± SD	81.2 ± 15.5	78.4 ± 16.3	1.295	0.197
Ideal Body Weight (kg), Mean ± SD*	63.4 ± 11.8	63.0 ± 10.5	0.305	0.776

**Table 2:** Clinical and Treatment Characteristics Chi-square tests are used for nominal data, and independent samples t-tests are used for continuous data MAP: Mean Arterial Pressure; RASS: Richmond Agitation Sedation Scale

Characteristic	Actual Body Weight (n = 98)	Adjusted Body Weight (n = 127)	Test Statistic	p-value
Baseline MAP (mmHg), Mean ± SD	87.0 ± 18.3	85.5 ± 15.8	0.670	0.503
Baseline Heart Rate (bpm), Mean ± SD	93.2 ± 21.6	93.1 ± 21.8	0.029	0.977
Baseline RASS Prior to Dexmedetomidine, n (%)			15.562	0.077
-5	2 (2.1)	1 (0.8)	-	
-4	4 (4.1)	3 (2.4)		
-3	8 (8.2)	2 (1.6)		
-2	11 (11.3)	11 (8.8)		
-1	20 (20.6)	34 (27.2)		
+0	11 (11.3)	22 (17.6)		
+1	21 (21.6)	26 (20.8)		
+2	12 (12.4)	22 (17.6)		
+3	4 (4.1)	4 (3.2)		
+4	4 (4.1)	0 (0)		
Mechanically Ventilated at Dexmedetomidine Initiation, n (%)	70 (72.2)	80 (64.5)	1.460	0.248
Coronary Artery Disease, n (%)	13 (13.3)	13 (10.2)	0.497	0.532
Sepsis, n (%)	18 (18.4)	23 (18.1)	0.002	1.000

Clinical outcomes

There were similar incidences of the primary outcomes of bradycardia and hypotension between the two groups (Table 3). Hypotension occurred in 71.4% of patients in the ABW group, compared to 69.3% in the AdjBW group (p = 0.770). Bradycardia occurred in 28.6% of patients in the ABW group, compared to 25.2% of patients in the AdjBW group (p = 0.649). The lowest recorded MAP was 60.4 ± 11.1 mmHg and 61.4 ± 12.0 mmHg in the ABW group and AdjBW group, respectively (p = 0.528). The lowest recorded heart rate was 61.2 ± 11.8 bpm and 65.2 ± 14.7 bpm in the ABW group and AdjBW group, respectively (p = 0.027, Wilcoxon rank sum p-value = 0.075).

**Table 3:** Study Outcomes *Independent sample median test Chi-square tests are used for nominal data, and independent samples t-tests are used for continuous data MAP: Mean Arterial Pressure

Characteristic	Actual Body Weight (n = 98)	Adjusted Body Weight (n = 127)	Test Statistic	p-value
Incidence of MAP <65, n (%)	70 (71.4)	88 (69.3)	0.121	0.770
Lowest Recorded MAP (mmHg), Mean ± SD	60.4 ± 11.1	61.4 ± 12.0	0.633	0.528
Incidence of Bradycardia, n (%)	28 (28.6)	32 (25.2)	0.322	0.649
Lowest Recorded Heart Rate (bpm), Mean ± SD	61.2 ± 11.8	65.2 ± 14.7	2.224	0.027
Incidence of Self-Extubation, n (%)	2 (2.9)	1 (1.3)	0.483	0.599
Addition of Another Sedative Infusion, n (%)	25 (25.5)	37 (29.1)	0.364	0.652
Hospital Length of Stay (Days), Median*	11.0	13.0	2.125	0.078

The secondary outcome of hospital length of stay was not statistically significant, with a median of 11 days in the ABW group compared to 13 days in the AdjBW group (p = 0.078). Additionally, the incidence of self-extubation was rare, occurring in 2.9% of patients in the ABW group and 1.3% of patients in the AdjBW group (p = 0.599). Patients required the addition of another sedative continuous infusion 25.5% of the time in the ABW group, compared to 29.1% of the time in the AdjBW group (p = 0.652).

## Discussion

Our retrospective cohort study found that dosing dexmedetomidine using AdjBW, compared to ABW, resulted in a lower incidence of hypotension and bradycardia, but this did not reach statistical significance. While the overall incidence of bradycardia was not statistically different between our study groups, there was a significant finding of the lowest recorded minimum heart rate in the ABW group compared to the AdjBW group.

Dexmedetomidine is a clinically important pharmacologic therapy used for short-term light to moderate sedation in critical care settings. Dexmedetomidine use confers the potential for the development of well-documented cardiovascular side effects, including hypotension and bradycardia, due to its α2-adrenoreceptor agonist-induced inhibition of norepinephrine [5]. With the high prevalence of comorbid obesity in critical care settings, a proportionally small number of studies have explored dexmedetomidine dosing considerations required in this patient population. Previous studies have shown that lower doses of dexmedetomidine in obese patients may be required, due to altered pharmacokinetics, to avoid adverse effects and supratherapeutic concentrations [2,3]. One study evaluated the safety and efficacy of weight-based sedation with dexmedetomidine and propofol in ICU patients with morbid obesity, and this study revealed that over-sedation was seen among all cohorts of patients, further reinforcing the need for additional studies in this patient population [8]. Atyia et al. previously established appropriate sedation maintenance in AdjBW compared to ABW dexmedetomidine dosing [1]. Additionally, the previously mentioned study included secondary outcomes, which found no statistically significant differences in the incidence of bradycardia between ABW and AdjBW groups, significantly lower median heart rate in the ABW group that developed bradycardia, and a significantly higher incidence of hypotension in the AdjBW group compared to the ABW [1]. The lack of literature that specifically addresses the cardiovascular impacts of dexmedetomidine dosing adjustments in the obese critically ill population calls for further primary investigation and validation of reports.

The findings in our study confirm existing literature regarding similar incidences of bradycardia between both groups for dosing dexmedetomidine in critically ill patients. This study additionally reinforces that dosing dexmedetomidine using ABW was associated with a significantly lower minimum heart rate compared to the AdjBW group. Our study differs from prior literature regarding similar incidences of hypotension between ABW and AdjBW dexmedetomidine dosing, as previous literature reported increased incidences of hypotension in the AdjBW dosing group [1]. The differences in these findings may be due to multiple factors. Our study population experienced a higher overall incidence of hypotension with dexmedetomidine use compared to previously published data. Previously reported hypotension incidence ranges from 49% to 59% [1]. Our study reports a 71.4% incidence of hypotension in the ABW group and 69.3% in the AdjBW group. Consideration of alternate definitions of hypotension between studies may also impact results and extrapolation of data to clinically significant organ hypoperfusion. Atyia et al. reported the use of a systolic blood pressure of less than 90 mmHg to define the pathology, as opposed to MAP of less than 65 mmHg, as used in our study [1].

Overall, AdjBW dosing of dexmedetomidine in obese critically ill patients appears to be safe, when compared to ABW dosing, based on favorable hemodynamic outcomes as demonstrated by our study.

There were several limitations to this study. The first was that this study was a retrospective chart review and utilized data originally collected during the course of clinical care at a single hospital system. Variability in clinical documentation and institutional documentation practices may have impacted this study. Another limitation of this study was the exclusion of dexmedetomidine dosing data. Dosing was not included due to the titratable nature of this medication within our healthcare system and the lack of ability to reproducibly examine the timing of dosing effects on hemodynamic outcomes. Similarly, the duration of dexmedetomidine use was also not included due to inconsistency between order and infusion end times. Higher doses between treatment groups may have contributed to an increased incidence of the primary outcome; however, this data was not assessed. Another limitation was that this study sample was derived from critically ill patients within a single healthcare system’s patient population and thus may not be representative of the general population, making it prone to sampling bias. ICU length of stay was not able to be accurately assessed due to exceeded ICU capacities during pandemic surges, when some critically ill patients were placed in non-critical care units. Lastly, this study included a relatively small sample size, which does not meet the pre-specified power criteria due to time restrictions, patients not meeting inclusion criteria, and missing data, all of which could have contributed to the results of this study. A random sample of patients was unable to be obtained due to the limited number of patients who met the inclusion and exclusion criteria.

## Conclusions

Similar incidences of hypotension and bradycardia were observed between both groups. A statistically significant lower recorded heart rate was observed in the ABW group compared to the AdjBW group. In conclusion, dosing dexmedetomidine using AdjBW instead of ABW appears to be safe in this patient population. Future prospective studies with larger patient populations, evaluation of dosing correlation, and examination of translated clinical implications on end-organ function should be considered to gain further insight into this clinical question.
